# Mapping the use of Group-Based Trajectory Modelling in medication adherence research: A scoping review protocol

**DOI:** 10.12688/hrbopenres.13056.2

**Published:** 2020-08-05

**Authors:** Caroline A. Walsh, Sara Mucherino, Valentina Orlando, Kathleen E. Bennett, Enrica Menditto, Caitriona Cahir

**Affiliations:** 1 Division of Population Health Sciences, Royal College of Surgeons in Ireland, Beaux Lane House, Lower Mercer's Street, Dublin 2, Dublin, D02 DH60, Ireland; 2 CIRFF, Centre for Pharmacoeconomics, University of Naples Federico II, Naples, Italy, Italy

**Keywords:** Medication Adherence, Patient Compliance, group-based trajectory modelling, scoping review, pharmacy refill claims, dispensing, longitudinal, trajectory analysis

## Abstract

The use of group-based trajectory modelling (GBTM) within the medication adherence literature is rapidly growing. Researchers are adopting enhanced methods to analyse and visualise dynamic behaviours, such as medication adherence, within ‘real-world’ populations. Application of GBTM based on longitudinal adherence behaviour allows for the identification of adherence trajectories or groups.  A group is conceptually thought of a collection of individuals who follow a similar pattern of adherence behaviour over a period of time. A common obstacle faced by researchers when implementing GBTM is deciding on the number of trajectory groups that may exist within a population. Decision-making can introduce subjectivity, as there is no ‘gold standard’ for model selection criteria.

This study aims to examine the extent and nature of existing evidence on the application of GBTM for medication adherence assessment, providing an overview of the different GBTM techniques used in the literature.

The methodological framework will consist of five stages: i) identify the research question(s); ii) identify relevant studies; iii) select studies; iv) chart the data and finally, v) collate, summarise and report the results. Original peer-reviewed articles, published in English, describing observational and interventional studies including both concepts and/or sub-concepts of GBTM and medication adherence or any other similar terms, will be included. The following databases will be queried: PubMed/MEDLINE; Embase (Ovid); SCOPUS; ISI Web of Science and PsychInfo. This scoping review will utilise the PRISMA extension for Scoping Reviews (PRISMA-ScR) tool to report results.

This scoping review will collect and schematise different techniques in the application of GBTM for medication adherence assessment available in the literature to date, identifying research and knowledge gaps in this area. This review can represent an important tool for future research, providing methodological support to researchers carrying out a group-based trajectory analysis to assess medication adherence in a real-world context.

## Introduction

Medication adherence is generally described as the process by which people take their medication as prescribed or as agreed with their prescriber. A taxonomy has been developed to describe the three distinct, yet, inter-related processes involved in medication adherence; initiation, implementation and discontinuation ^1^. Initiation adherence refers to the first prescription for the medication being dispensed in the pharmacy. The implementation phase refers to the execution of the recommended dosing regimen; skipping doses, delaying refills or taking drug holidays are examples of implementation non-adherence. Discontinuation occurs when the patient stops taking the medication, thus beginning a period of non-persistence. Persistence is another term that is commonly used and refers to the duration the patient takes the medication for, encompassing the initiation and implementation phases ^1^. Initiation, discontinuation and persistence are usually modelled as time-to-event phenomena, whereas implementation adherence can be reported in a variety of ways, usually involving summary statistical estimates ^2^. Summary adherence estimates include measures such as the proportion of days covered (PDC) and medication possession ratio (MPR), which are commonly used to describe adherence using administrative claims data ^2^.

However, adherence is a dynamic behaviour, potentially varying over time due to a number of factors. It has been suggested that longitudinal methods should be used to analyse implementation adherence, as aggregating behaviours over time into a single summary estimate of adherence which is then dichotomized can result in a loss of information about the detailed patterns of adherence ^1, 3^. This is of particular concern for the estimation of adherence to medications used to treat long-term, chronic conditions. Using summary measures can often lead to difficulty in estimation of the time point or phase at which non-adherence is likely to occur in a population. Indeed, two individuals may have the same average adherence value over a period of time (i.e. 50%) but one may skip doses regularly, whereas the other may have had high initial adherence followed by a long gap in dispensing ^3^. Over the past number of years, group-based trajectory modelling (GBTM) has become more frequently applied in adherence research, to enhance understanding of adherence behaviours ^3^. This is due to the availability of freely available, downloadable software programmes that can be used within existing statistical packages to implement GBTM ^4^. Indeed, GBTM has been recently applied to older Irish populations to identify trajectories based on adherence to anti-hypertensive medications ^5, 6^ and across multimorbidity ^7, 8^. Identification of groups vulnerable to poor adherence and the time-point at which this can occur in the treatment process may help to inform targeting of medication management interventions.

GBTM is a type of finite mixture modelling which uses trajectory groups to estimate an unknown distribution of trajectories that exist within a population ^9^. The groups identified by the models should not be thought of as literal entities, but rather as discrete groups that may exist within a population ^9^. Considering the application of GBTM within adherence research, a group is conceptually thought of as a collection of individuals who follow approximately the same pattern of adherence behaviour, equivalent to a contour line on a contour map ^9^. GBTM is operationalised by repeatedly measuring adherence at frequent time intervals (i.e. monthly) and grouping individuals with similar longitudinal adherence patterns ^10^. GBTM may aid the precise identification of the timing of transition from one adherence phase to another, namely movement from the implementation phase into the discontinuation phase. The model assumes that within-person correlation is explained completely by the adherence trajectory curve estimated for each person’s group ^10^. Model parameters are estimated using maximum likelihood ^4^, meaning unbiased estimates can be produced in the presence of missing data, provided such data are missing at random ^9^.

A common obstacle faced by researchers when implementing GBTM is deciding on the number of trajectory groups that may exist within a population. Prior to conducting any statistical analysis, the maximum number of groups likely to exist based on the size of the population and existing evidence is estimated. However, adherence is often reported as a dichotomous variable in the literature, resulting in participants being classified into two adherence groups; adherent and non-adherent. The threshold most commonly used to determine this classification has been arbitrarily set at 80% ^3, 11^, originating from anti-hypertensive medication studies ^12, 13^, with little validation across other conditions. Therefore, *a priori* theories on the number, shape and size of adherence trajectory groups are often absent ^9^.

Determining the number of optimum number of adherence groups that hypothetically exist within a population is based on statistical fit indices, most commonly the Bayesian Information Criteria (BIC) ^14^, Akaike Information Criteria (AIC) ^15^, Lo-Mendall-Rubin likelihood ratio test (LMR-LRT) ^16^ and entropy ^9^. BIC and AIC aim to identify the most parsimonious model by balancing model complexity versus goodness to fit to the study data ^9^. Lower index values indicate improved model fit. The LMR-LRT utilises a likelihood-ratio-based approach, helping to determine the optimum model between ‘k-1’ and k class models; a p value >0.05 is used to reject a new model class containing an additional group (k) ^9, 17^. Entropy is used to measure how accurately the model classifies participants into different trajectories or groups. The average posterior probabilities of group membership are calculated with values closer to 1 indicating greater precision. Previous adherence GBTM studies have used thresholds of probability ≥0.70 to indicate presence of sufficient entropy in a model ^7, 17^, whereas others did not use explicit cut-offs ^5, 6, 10^.

### Rationale

To date, and to the best of our knowledge, there is no existing peer-reviewed or published synthesis of the use of GBTM in medication adherence research. As the popularity of GBTM is growing in the adherence literature, it is necessary to map the evidence within this area, to help summarise existing evidence and guide future research. A scoping review methodology is used for such a mapping exercise as it is suited to broad research questions and is useful in fields such as adherence measurement, where there is a lot of measurement heterogeneity ^18^. Scoping reviews not only highlight the extent of research available on a topic, but also allow for a description of the conduct of such research ^18^. A synthesis of the literature of the use of GBTM in medication adherence measurement will help to identify research deficits and knowledge gaps in this area, informing future research ^18– 20^.

### Objective and aims

The objective of this scoping review is to describe the nature, number, scope and methodology of published research articles using group-based trajectory modelling to measure medication adherence and to identify what further research is required.

Specifically, we will aim to:

- Systematically explore the extent of relevant empirical literature on the use of group-based trajectory analysis applied to medication adherence in longitudinal studies.
- Map and categorise publications obtained according to the following taxonomy: purpose of study (identify adherence behaviours, groups for intervention targeting), model selection criteria used to determine adherence groups, and outcomes typology (validation against clinical/other health outcomes or absent).
- Provide an overview of the different GBTM techniques used for medication adherence measurement in the literature and guidance for future adherence research.

## Methods

The methodological framework for conducting this scoping review was informed by published guidance ^18– 20^. This process consists of five different stages. ^19^: (1) identify the research question(s); (2) identify relevant studies; (3) select studies; (4) chart the data and (5) collate, summarise and report the results. There is an optional sixth stage, ‘consultation with relevant stakeholders’ that may be prioritised in social science research ^20^. However, the relevance of this stage in the current scoping review is not apparent, and as such we will not be formally engaging with external stakeholders prior to completion of the scoping review.

In order to provide a descriptive account of the status of GBTM in adherence research and identify knowledge gaps, a scoping review of the literature is most appropriate. Findings from the review may help to promote standardisation of GBTM methodology in future adherence studies. As in the case of systematic reviews, scoping reviews also use a systematic approach to research, screening and reporting.

### Identifying the research question

There has been an increasing use of GBTM as a tool for longitudinal adherence measurement and visualisation; however, there appears to be a lack of standardisation in the methodological approach similar to the existing heterogeneity in medication adherence measurement ^21, 22^. The lack of standardisation can introduce varying degrees of subjectivity into the decision-making process required with application of GBTM, limiting comparison across studies. While some degree of subjectivity may be necessary ^23^, it would be advantageous to summarise the various approaches used to help inform future research. The following research questions were identified for the review based on the aims of the review:

1. What is main purpose of application of GBTM in medication adherence research?
2. What is the range of statistical techniques employed to apply GBTM for the measurement of medication adherence in the literature?
3. Which clinical or other outcomes been used to validate the use of GBTM in medication adherence research?
4. Is the use of GBTM for measurement of medication adherence prominent in specific populations or cohorts? Are there differences in methodological approaches consequentially?
5. Are there recommendations for the standardisation of GBTM techniques within adherence research?
6. What are the current knowledge gaps relating to the application of GBTM in medication adherence research that require further research?

Identification of additional research questions may be an iterative process, informed by emerging themes that appear while conducting the scoping review.

### Identifying relevant studies

***Inclusion criteria.*** Peer-reviewed publications of empirical research which apply GBTM to the measurement of medication adherence will be considered for inclusion. Furthermore, publications will have to include in their abstract both concepts and/or sub-concepts of group-based trajectory modelling or any other similar term (e.g., group-based analysis, trajectory model) and medication adherence.

Original articles, published in English, describing observational studies will be included. No restrictions will be placed on study design (case-control, cohort, prospective, retrospective etc), although it is unlikely cross-sectional studies will be suitable, given the need for longitudinal data to perform GBTM. Randomised controlled trials will be included if it is specified in the study abstract that longitudinal analysis was performed as part of the study.

In the first instance, no limitations will be applied in the year of publication, therefore, all studies in the literature to date will be identified. However, if excessive search results are identified after de-duplication (>4000), search results will be narrowed to articles published after January 2005, as it is after this time that GBTM mainly emerged in the medication adherence field.

***Exclusion criteria.*** Only articles available in English will be included. Furthermore, grey literature including guidelines, booklets, reports, and clinical guidelines will not be included. Unpublished academic documents such as theses and dissertations will be not included in the scoping review. In addition, conference abstracts will not be considered as the purpose of this scoping review is to extract data relating to the methodological approach used in GBTM studies, of which abstracts provide limited detail. Similarly, study protocols will be excluded as hypothetical analytic approaches may differ from actual methodological approaches applied. However, we will attempt to contact authors of relevant protocols and abstracts to ascertain the availability of full research reports, if not identified by the existing search. Systematic and literature reviews will not be included in the review, but instead, will be used to identify potentially relevant observational studies.

***Information sources and search strategy.*** For the present scoping review, the identification of relevant studies will be achieved by searching electronic databases of the published literature, which will include the following: Medical Literature Analysis and Retrieval System Online (PubMed/MEDLINE); Embase (Ovid); SCOPUS; ISI Web of Science and PsychInfo. A comprehensive search strategy has been developed with the assistance of a medical librarian, to identify relevant studies. Search terms were determined by team members and further developed after consultation with the medical librarian. Search strings combined keywords, phrases and Medical Subject Headings (or equivalent) across two concepts using the AND Boolean operator: (1) medication adherence; (2) group-based trajectory modelling. Terms for medication adherence were informed by a previous systematic review involving some of the authors ^22^,and expanded upon. Search terms relating to ‘medication adherence’ include patient compliance, treatment adherence, medication (non-) compliance, medication persistence as well as the phases of medication adherence as per the ABC taxonomy (initiation, implementation, discontinuation) ^1^. For ‘group-based trajectory modelling’, related terms include ‘gbtm’, ‘trajectory analysis’, ‘longitudinal trajectory’, ‘latent class analysis’ and ‘adherence pattern’. Within each concept, relevant terms were combined using the ‘OR’ Boolean operator. The search strategy was developed for use in PubMed/Medline and will be further adapted for use across the four other electronic databases. The full search strategy will be included in the final manuscript. Electronic databases will be searched from inception, with no limitations or filters placed on records obtained, until acceptance for publication.

Further, a citation search of included full-texts will be undertaken in Google Scholar to identify relevant published studies that were not retrieved through database searching.

### Selecting relevant studies

The search results will be downloaded to an electronic referencing system and duplicates removed. As stated previously, should an excessive number of independent records be retrieved, records will be limited to those published during or after 2005. One author (CW) will independently screen the title and abstracts of all retrieved articles for studies that use GBTM to measure medication adherence. A second reviewer (SM) will independently screen a 50% random sample of abstracts. Abstracts that are deemed unsuitable for progression to full-text review will be allocated to folders citing the reason for exclusion. Once each reviewer has selected relevant records for full-text review independently, results will be compared between reviewers and discussions held until consensus is reached. The second reviewer will review the abstracts, excluded from their random sample, that were selected for full text review by the main reviewer. If a conflict remains following discussions, a third reviewer (CC or EM) will be consulted until consensus is reached. Two reviewers (SM and CW) will review each full text independently, citing reasons for exclusion if not deemed suitable for inclusion in the scoping review. Discussions will be held until consensus is reached, adhering to the inclusion and exclusion criteria specified *a priori*. Similar to the abstract screening process, a third reviewer will be consulted (CC or EM) to resolve any conflict. Reasons for exclusion of texts after full-text review will be documented and reported in the PRISMA study flow diagram.

### Charting the data

A standardised data charting form was created in Excel *a priori*, based on guidance pertaining to data charting in scoping reviews from the Joanna Brigg’s Institute Reviewer’s Manual ^24^. We have updated the form based on useful suggestions provided by protocol reviewers. Initial categories included general study characteristics such as authors, title, DOI, year of publication and country. Next, information on the study design will be collected including the aims/purpose of the study, whether adherence was modelled as an exposure, covariate or outcome, descriptives of the study population and sample size (e.g. age, gender, and ethnicity) and the medication or disease group studied. Further, information specific to medication adherence measurement will be collected including the data source, duration of adherence measurement (length of observation), the time intervals used, the GBTM method applied (statistical package used), the maximum number of adherence trajectories selected, along with the evidence base used to inform this number, if applicable, and finally, the model selection criteria used to select the optimum number of adherence trajectories. Information on the order used (cubic, quadratic etc) to model groups will be extracted, if available. Lastly, information pertaining to results and findings from the study will be extracted, including the number of adherence trajectories identified, details relating to validation against clinical outcomes, if applicable, and any adjustment for covariates and limitations of the study. Initially, the data charting form will be piloted using two or three relevant studies identified from database searches. This will be done independently by two reviewers (SM and CW) and discussions will be held between the two reviewers following this to identify additional data that needs to be charted, along with amendments of existing headings if required ^19, 20^. Study authors will be contacted if further clarification is required in relation to data extracted.

### Collating, summarising and reporting the results

This scoping review will utilise the PRISMA extension for Scoping Reviews (PRISMA-ScR) tool ^25^. A flow diagram will be used to outline the selection of data sources, including descriptive reasons for exclusion at the full-text review stage. Characteristics of the included studies will be described based on the descriptive headings in the data extraction form. Specifically, the evidence will be summarised and reported using the taxonomy described in the aims; purpose of the study, model selection criteria use and outcomes typology (if applicable). Guided by the research questions, additional headings will be used to summarize the studies if findings are not sufficiently communicated using the aforementioned taxonomy. For instance, it may be possible to categorise studies based on their study population (paediatric vs older people) or disease area (cardiovascular, musculoskeletal etc). Formal quality appraisal of included studies will not be undertaken, as the aim of scoping reviews is to provide an overview of the existing evidence base regardless of quality ^18^. A general interpretation of the evidence will be provided, as well as identification of potential knowledge gaps. The strengths and limitations of the scoping review will be outlined, as well as potential guidance for future research in the final report. Any deviations from this protocol, including reasons, will also be detailed.

## Conclusion

The over-arching purpose of GBTM is to identify discrete groups that have meaningful differences in terms of pre-existing characteristics or subsequent outcomes or treatment response. If the groups or trajectories cannot be distinguished on the basis of such dimensions, identification of different trajectories serves little purpose ^9^. This scoping review will collect and schematize different techniques in the application of group-based trajectory modelling for medication adherence assessment available in literature to date. The main expectation of the exploration of the literature will be to summarise evidence and identify research and knowledge gaps in this area to inform future research. Indeed, recent studies have called for greater transparency over the subjective decisions involved in applying GBTM for medication adherence assessment ^23^. This review may represent an important tool for future research, in order to methodologically support researchers who will carrying out group-based trajectory analysis to assess medication adherence in real-world contexts.

## Data availability

No data are associated with this article.
