# Supplementary material for: Mapping the use of Group-Based Trajectory Modelling in medication adherence research: A scoping review protocol
Source: HRB Open Res. 2020 Aug 5;3:25. Originally published 2020 May 19. [Version 2] doi: 10.12688/hrbopenres.13056.2 (PMC7265572; doi:10.12688/hrbopenres.13056.2)
Supplement: Peer review history

# Peer review: Reviewer response for version 2

**DOI:** 10.21956/hrbopenres.14228.r27774

**Published:** 2020-08-06

**Authors:** Ryan P. Hickson

**Affiliations:** 1 Division of Pharmaceutical Outcomes & Policy, UNC Eshelman School of Pharmacy, University of North Carolina at Chapel Hill, Chapel Hill, NC, USA

Thank you for you thoughtful responses to my comments. Looking forward to when this review is complete. I do not require any new changes, but would like to state I still find the definition of entropy confusing at the end of the introduction. I have seen papers use the maximum posterior probabilities themselves to evaluate GBTM model fit (the distribution of posterior probabilities for each group are evaluated, so if you have 4 groups, you are evaluating 4 separate averages), and I have seen papers use entropy - which uses posterior probabilities in its calculation - to evaluate GBTM model fit (a single entropy estimate is provided for the full model, so no matter how many groups are in the model, you are only evaluating 1 entropy estimate for model fit). I think equations 12 and 13 and the following paragraphs help illustrate this distinction in this paper ^1^. I only bring this up as it seems these concepts are not clearly explained/understood in all of the medication adherence GBTM papers I have read.

Is the study design appropriate for the research question?

Yes

Is the rationale for, and objectives of, the study clearly described?

Yes

Are sufficient details of the methods provided to allow replication by others?

Yes

Are the datasets clearly presented in a useable and accessible format?

Not applicable

Reviewer Expertise:

Medication adherence, pharmacoepidemiology, population health science, causal inference, cardiometabolic conditions.

I confirm that I have read this submission and believe that I have an appropriate level of expertise to confirm that it is of an acceptable scientific standard.

## References

[1] : An overview of mixture modelling for latent evolutions in longitudinal data: Modelling approaches, fit statistics and software. *Advances in Life Course Research*.2020;43: 10.1016/j.alcr.2019.100323 10.1016/j.alcr.2019.100323 36726256

# Peer review: Reviewer response for version 2

**DOI:** 10.21956/hrbopenres.14228.r27773

**Published:** 2020-08-05

**Authors:** Hannah Durand

**Affiliations:** 1 School of Psychology, National University of Ireland, Galway, Ireland

I have no further comments to make.

Is the study design appropriate for the research question?

Yes

Is the rationale for, and objectives of, the study clearly described?

Yes

Are sufficient details of the methods provided to allow replication by others?

Yes

Are the datasets clearly presented in a useable and accessible format?

Not applicable

Reviewer Expertise:

Medication adherence; quantitative methods; evidence synthesis.

I confirm that I have read this submission and believe that I have an appropriate level of expertise to confirm that it is of an acceptable scientific standard.

# Peer review: Reviewer response for version 1

**DOI:** 10.21956/hrbopenres.14152.r27425

**Published:** 2020-06-09

**Authors:** Ryan P. Hickson

**Affiliations:** 1 Division of Pharmaceutical Outcomes & Policy, UNC Eshelman School of Pharmacy, University of North Carolina at Chapel Hill, Chapel Hill, NC, USA

**Paper summary and general comments**

 This paper is a protocol describing a proposed scoping review of the use of group-based trajectory modelling (GBTM) in the medication adherence literature. The rationale for this review is strong, and once completed, this review should be of great interest to medication adherence researchers, especially those wishing to use a GBTM analysis. Particularly, the identification of relevant studies, search strategy, selecting relevant studies, data charting, and collating are well described. This makes the search strategy reproducible for others and will help readers easily understand the scope of what was reviewed. My only concern is that the rationale for excluding any potential randomized studies that use GBTM to assess medication adherence does not seem justified.

**Specific comments**

Major

 Introduction, paragraph 4: This paragraph is unclear to me. You seem to be talking about 2 different concepts: (1) how many latent groups to use for the GBTM, and (2) whether to use continuous adherence measures (ranging from 0% to 100%) or binary adherence measures (e.g. an 80% cutoff) which would then be modeled as a continuous GBTM (CNORM distribution) or a binary GBTM (LOGIT distribution), respectively. Consider revising.

 Introduction, paragraph 5: I believe your use of “entropy” and “posterior probabilities” is confusing and misleading here. Each individual is assigned a posterior probability of belonging to each of the groups in the GBTM. The average maximum posterior probability (averaging the posterior probabilities within each group, but only including posterior probabilities for individuals assigned to the given group) is one method used to assess the model fit ^1^. Entropy and relative entropy are also measures of model fit that use posterior probabilities in its calculation, but it is not a simple average of posterior probabilities; only relative entropy is bound between 0 and 1 ^2^

 Exclusion criteria. I doubt you will find many if any randomized trials using GBTM for medication adherence; however, I would recommend against excluding RCTs outright. Patients would not be randomized to adherence groups; GBTMs in this setting may not be “real-world” because it is in a restricted population eligible for an RCT; however, GBTM is still measuring the “real-world” medication-taking behavior for patients assigned to a given intervention.

 Charting the data: “duration of adherence measurement”. Does this refer to how long adherence was measured (e.g. 6 months vs. 12 months)? Or to the time-intervals in which adherence was measured (e.g. 30 1-day intervals for 1 month of longitudinal adherence; 6 30-day intervals for 6 months of longitudinal adherence)? Both of these concepts would be important.

Minor

 General: consider reviewing paper for minor typos, etc. to improve readability.

 Abstract: “This study aims to examine the extent and nature…” It is not clear what this means, consider revising.

 Introduction, end of paragraph 1: “… usually involving summary statistical estimates”. This is unclear. Perhaps an example would help clarify.

 Introduction, paragraph 3: you state that GBTM has been used for the past 15 years. Are you sure this is accurate? I had thought Franklin *et al*. was the first to use GBTM for medication adherence in 2013 ^3^, but the review you cite ^4^ shows an earlier paper by Modi *et al.* in 2011 that used GBTM for medication adherence ^5^. However, I do not see or know of any GBTM papers for medication adherence that were published before this.

 Rationale: “mapping” and “scoping review” may be standard terminology for this type of review, but consider giving a clearer definition of these terms earlier in the paper. Readers of your final paper, specifically researchers who want to use GBTM for medication adherence projects, may not be familiar with these terminologies.

 Objective and aims: What do you mean by “nature” and “extent”?

 Methods, paragraph 1 and throughout. For stage (1), “identify the research question”, I believe clarification is in order. May be difficult for reader to understand if this means research questions as it relates to your review, or the research questions of the papers you are reviewing.

 Search strategy. I believe the search terms are fairly well-defined, but a table may help to better visualize all the possible combinations and how the Boolean operators are applied. Additionally, for medication adherence search terms, what about the terms “adherence” and “compliance”, etc., without the word medication directly in front of it? Would you potentially be missing some papers who never explicitly said “medication adherence”? What about wildcards? E.g. adheren* to capture adherence and adherent?

 Charting the data: What about how medication adherence was modeled as a function of time? All cubic? All quadratic? A mixture of 0-order through quartic? ^6^

 Charting the data: It is unclear if this is mentioned elsewhere, but what about the purpose of the GBTM. Are the groups meant to be an exposure, with associations with clinical outcomes the purpose? Are the adherence groups the outcome of the study, to understand patterns of adherence in normal practice or as part of an intervention to change adherence? Or are the groups meant to adjust for health-seeking behavior? Other potential purposes….

 Conclusion: “The over-arching purpose…” Are these the only reasons to use GBTM for medication adherence studies? What about measuring medication adherence simply to observe what the behavior is? Or as a health-seeking behavior to adjust for the healthy user/sick stopper bias?

Is the study design appropriate for the research question?

Yes

Is the rationale for, and objectives of, the study clearly described?

Yes

Are sufficient details of the methods provided to allow replication by others?

Yes

Are the datasets clearly presented in a useable and accessible format?

Not applicable

Reviewer Expertise:

Medication adherence, pharmacoepidemiology, population health science, causal inference, cardiometabolic conditions.

I confirm that I have read this submission and believe that I have an appropriate level of expertise to confirm that it is of an acceptable scientific standard.

## References

[1] : Group‐based modeling of development. *Harvard University Press*.2005;

[2] : Structural equation modeling: Applications using Mplus. *Wiley*.

[3] : Group-based trajectory models: a new approach to classifying and predicting long-term medication adherence. *Med Care*.2013;51(9) : 10.1097/MLR.0b013e3182984c1f 789-96 10.1097/MLR.0b013e3182984c1f 23685406

[4] : The myths of medication adherence. *Pharmacoepidemiol Drug Saf*.26(12) : 10.1002/pds.4334 1437-1441 10.1002/pds.4334 28994158

[5] : Patterns of nonadherence to antiepileptic drug therapy in children with newly diagnosed epilepsy. *JAMA*.2011;305(16) : 10.1001/jama.2011.506 1669-76 10.1001/jama.2011.506 21521848PMC3480079

[6] : Opening the black box of the group-based trajectory modeling process to analyze medication adherence patterns: An example using real-world statin adherence data. *Pharmacoepidemiol Drug Saf*.2020;29(3) : 10.1002/pds.4917 357-362 10.1002/pds.4917 31802581PMC7058496

# Response

**Published:** 2020-07-25

**Authors:** Caroline Walsh

**Affiliations:** Royal College of Surgeons, Ireland

*Paper summary and general comments*

*This paper is a protocol describing a proposed scoping review of the use of group-based trajectory modelling (GBTM) in the medication adherence literature. The rationale for this review is strong, and once completed, this review should be of great interest to medication adherence researchers, especially those wishing to use a GBTM analysis. Particularly, the identification of relevant studies, search strategy, selecting relevant studies, data charting, and collating are well described. This makes the search strategy reproducible for others and will help readers easily understand the scope of what was reviewed. My only concern is that the rationale for excluding any potential randomized studies that use GBTM to assess medication adherence does not seem justified.*

Response:

Thank you for your suggestions. We have responded to specific comments below and in the manuscript as indicated.

*Specific comments*

*Major*

*Introduction, paragraph 4: This paragraph is unclear to me. You seem to be talking about 2 different concepts: (1) how many latent groups to use for the GBTM, and (2) whether to use continuous adherence measures (ranging from 0% to 100%) or binary adherence measures (e.g. an 80% cutoff) which would then be modeled as a continuous GBTM (CNORM distribution) or a binary GBTM (LOGIT distribution), respectively. Consider revising.*

Response:

Thank you. The review is about the number of trajectories to use for the GBTM. We are not referring to the nature of the indicator adherence variable (continuous vs dichotomous) to be used in the GBTM and we do not mention that here. To clarify we have added ‘in the literature’ to the following sentence: ‘However, adherence is often reported as a dichotomous variable in the literature’

In adherence studies we often get 2 exposure/outcome groups based on dichotomisation of the adherence variable; adherent and non-adherent. As such, it can be difficult to specify a priori the maximum number of adherence groups that might exist within a certain population, unless GBTM has been previously carried out in a similar population or medication group.

*Introduction, paragraph 5: I believe your use of “entropy” and “posterior probabilities” is confusing and misleading here. Each individual is assigned a posterior probability of belonging to each of the groups in the GBTM. The average maximum posterior probability (averaging the posterior probabilities within each group, but only including posterior probabilities for individuals assigned to the given group) is one method used to assess the model fit1. Entropy and relative entropy are also measures of model fit that use posterior probabilities in its calculation, but it is not a simple average of posterior probabilities; only relative entropy is bound between 0 and 12*

Response:

Thank you. In a 2010 article from Nagin and Odgers(1), the following is stated in relation to entropy; ‘ Entropy is also used in model selection, as it indexes classification accuracy by averaging the posterior probabilities after individuals have been assigned to their most likely class, with values closer to 1 indexing greater precision (range 0 to 1).’ Having a minimum proportion (usually 5%) within each group is also used as a measured of model fit. We have added the following to the manuscript: ‘Estimation of the average posterior probabilities of group membership is included in the entropy calculation.’

*Exclusion criteria. I doubt you will find many if any randomized trials using GBTM for medication adherence; however, I would recommend against excluding RCTs outright. Patients would not be randomized to adherence groups; GBTMs in this setting may not be “real-world” because it is in a restricted population eligible for an RCT; however, GBTM is still measuring the “real-world” medication-taking behavior for patients assigned to a given intervention.*

Response:

We have modified the criteria as outlined in the reviewer response previously.

*Charting the data: “duration of adherence measurement”. Does this refer to how long adherence was measured (e.g. 6 months vs. 12 months)? Or to the time-intervals in which adherence was measured (e.g. 30 1-day intervals for 1 month of longitudinal adherence; 6 30-day intervals for 6 months of longitudinal adherence)? Both of these concepts would be important.*

Response:

Thank you for highlighting this omission and we agree. We think both length and time intervals are important data to extract from studies. We have amended the ‘charting the data’ section to state as follows: ‘Further, information specific to medication adherence measurement ….duration of adherence measurement (length of observation), the time intervals used…..’

*Minor*

*General: consider reviewing paper for minor typos, etc. to improve readability.*

Response:

Thank you.

*Abstract: “This study aims to examine the extent and nature…” It is not clear what this means, consider revising.*

Response:

Extent refers to how widespread the use of GBTM is in adherence research and nature refers to how it is used, what questions it aims to answer, which is what this review aims to examine.  These are broad terms which align with the purpose of scoping reviews-to describe the research area as opposed to in a systematic review which aims to answer a specific question. We have clarified that extent refers to the number or breadth of papers that use GBTM in medication research in the Objectives setting.

*Introduction, end of paragraph 1: “… usually involving summary statistical estimates”. This is unclear. Perhaps an example would help clarify.*

Response:

Thank you, we have amended the end of this paragraph to the following: ‘Summary adherence estimates include measures such as the proportion of days covered (PDC) and medication possession ratio (MPR), which are commonly used to describe adherence using administrative claims data. ^2^’

*Introduction, paragraph 3: you state that GBTM has been used for the past 15 years. Are you sure this is accurate? I had thought Franklin et al. was the first to use GBTM for medication adherence in 20133, but the review you cite4 shows an earlier paper by Modi et al. in 2011 that used GBTM for medication adherence5. However, I do not see or know of any GBTM papers for medication adherence that were published before this.*

Response:

Thank you for highlighting this. This is a general statement regarding the time period for which one gets publication hits in PubMed using the search strategy, but it not a definitive fact. As such we have modified the language here to say ‘Over the past number of years…’. We hope the review will provide more insight into when GBTM was first used in adherence research because as you have stated, there is evidence to suggest use prior to Franklin’s seminal paper in 2013(2).

*Rationale: “mapping” and “scoping review” may be standard terminology for this type of review, but consider giving a clearer definition of these terms earlier in the paper. Readers of your final paper, specifically researchers who want to use GBTM for medication adherence projects, may not be familiar with these terminologies.*

Response:

In the objective and aims section we state the following, which describes the purpose of the scoping review: ‘The objective of this scoping review is to describe the nature, number, scope and methodology of published research articles using group-based trajectory modelling….’. As per our response above, a scoping review is different to a systematic review in that it doesn’t aim to answer a specific question.

*Objective and aims: What do you mean by “nature” and “extent”?*

Response:

As responded to above.

*Methods, paragraph 1 and throughout. For stage (1), “identify the research question”, I believe clarification is in order. May be difficult for reader to understand if this means research questions as it relates to your review, or the research questions of the papers you are reviewing.*

Response:

The research question relates to the research question of the review, which is used to direct the search for studies, data extraction etc. We clearly state in the methods section that identification of the research question is the first stage in the scoping review process. To enhance clarity we have amended the ‘identifying the research question’ paragraph to state the following: ‘The following research questions for the review were identified based on the aims of the review:’

Search strategy. I believe the search terms are fairly well-defined, but a table may help to better visualize all the possible combinations and how the Boolean operators are applied. Additionally, for medication adherence search terms, what about the terms “adherence” and “compliance”, etc., without the word medication directly in front of it? Would you potentially be missing some papers who never explicitly said “medication adherence”? What about wildcards? E.g. adheren* to capture adherence and adherent?

Response:

We agree and we contemplated this. However, the current search strategy identified the papers we are aware of that use GBTM in medication adherence research, in addition to 1000’s of other potentially relevant papers (total results>6000). However, as outlined in the previous response to reviewer 2, there is a trade-off between comprehensiveness of the search strategy (particularly when it comes to medication adherence studies) and the resources required to search through the additional 1000’s of abstracts that broader terms can result in. In Scopus and Embase we have modified search terms including ‘medication adherence’ such that we obtain results that have medication and adherence in close proximity, but not necessarily together.

*Charting the data: What about how medication adherence was modeled as a function of time? All cubic? All quadratic? A mixture of 0-order through quartic?6*

Response:

Thank you for this suggestion, we agree this information would be useful to know. As such we have modified the data extraction section as follows: ‘Information on the order used (cubic, quadratic etc) to model groups will be extracted, if available.’

*Charting the data: It is unclear if this is mentioned elsewhere, but what about the purpose of the GBTM. Are the groups meant to be an exposure, with associations with clinical outcomes the purpose? Are the adherence groups the outcome of the study, to understand patterns of adherence in normal practice or as part of an intervention to change adherence? Or are the groups meant to adjust for health-seeking behavior? Other potential purposes….*

Response:

Thank you. Yes we agree and have stated the following in the charting the data section: ‘Next, information on the study design will be collected including the aims/purpose of the study, whether adherence was modelled as an exposure, covariate or outcome….’

*Conclusion: “The over-arching purpose…” Are these the only reasons to use GBTM for medication adherence studies? What about measuring medication adherence simply to observe what the behavior is? Or as a health-seeking behavior to adjust for the healthy user/sick stopper bias?*

Response:

The most useful application of GBTM in adherence research, from our point of view as pharmacists, psychologists and adherence researchers, is the more precise identification of the groups of people who may benefit from adherence interventions and the stage of treatment at which intervention would be most beneficial. For researchers who lack the assistance of programming experts, the computational workload required to carry out GBTM is larger in comparison to calculation of summary statistics. We agree that is interesting to observe adherence behaviour using this methodology but if it doesn’t provide additional insight over traditional metrics that can be actioned upon, the clinical relevance of its application is questionable. We hope the scoping review will help to provide some clarity on this.

References

1.            Nagin DS, Odgers CL. Group-based trajectory modeling in clinical research. Ann Rev Clin Psycholual review of clinical psychology. 2010;6:109-38. 10.1146/annurev.clinpsy.121208.131413

2.            Franklin JM, Shrank WH, Pakes J, Sanfélix-Gimeno G, Matlin OS, Brennan TA, et al. Group-based trajectory models: a new approach to classifying and predicting long-term medication adherence. Medical Ccare. 2013;51(9):789-96.

# Peer review: Reviewer response for version 1

**DOI:** 10.21956/hrbopenres.14152.r27428

**Published:** 2020-06-01

**Authors:** Julie Lauffenburger

**Affiliations:** 1 Center for Healthcare Delivery Sciences, Department of Medicine, Brigham and Women’s Hospital, Boston, MA, USA

Group-based trajectory modelling has been applied to the field of medication adherence since 2012, and as the authors point out, there has been heterogeneity in how the field has used these techniques. This is an interesting protocol, and the study, when completed, should provide insight to the field of medication adherence and the GBTM approaches.

 To enhance their study, I have a few minor suggestions for the authors:

- In the inclusion criteria, please clarify what terms will be used for group-based trajectory modeling and medication adherence. Both have many synonyms in the literature, and it would be useful for replicability to know which terms the authors used. It is not clear as presented whether this is a completely exhaustive list of the terms used.
- The authors should also clarify which data sources for trajectory modeling are being used in the underlying studies, such as whether they are all done using pharmacy refill/claims data.
- While I am not aware of any RCT that has used GBTM in its modeling for adherence to date, I would not have excluded them outright. The authors should at a minimum provide how many studies were excluded because they were RCTs.
- Similarly, it would also be useful to classify whether GBTM has exclusively been used as the way of classifying adherence as an outcome, or whether it has been applied (and how it has been applied) as an exposure or covariate.

Is the study design appropriate for the research question?

Yes

Is the rationale for, and objectives of, the study clearly described?

Yes

Are sufficient details of the methods provided to allow replication by others?

Yes

Are the datasets clearly presented in a useable and accessible format?

Not applicable

Reviewer Expertise:

Medication adherence, pragmatic trials, cardiometabolic disease, pharmacoepidemiology.

I confirm that I have read this submission and believe that I have an appropriate level of expertise to confirm that it is of an acceptable scientific standard.

# Response

**Published:** 2020-07-25

**Authors:** Caroline Walsh

**Affiliations:** Royal College of Surgeons, Ireland

*In the inclusion criteria, please clarify what terms will be used for group-based trajectory modeling and medication adherence. Both have many synonyms in the literature, and it would be useful for replicability to know which terms the authors used. It is not clear as presented whether this is a completely exhaustive list of the terms used.*

Response:

Thank you and we agree. We intend to publish the full search string used across all databases with the final manuscript. Given the numerous terms used for both medication adherence and group-based trajectory modelling it is unlikely that the list is completely exhaustive. However, initial searches have indicated >6000 search results for abstract screening from the current combined search strings across the databases. This search has identified well-known papers that have described the use of group-based trajectory modelling. In addition, we will be searching the reference lists of included texts in the review which should help to broaden the search.

*The authors should also clarify which data sources for trajectory modeling are being used in the underlying studies, such as whether they are all done using pharmacy refill/claims data.*

Response:

We agree this is an important piece of information to extract from the studies and have stated in the ‘charting the data’ section: ‘Further, information specific to medication adherence measurement will be collected including the data source..’.

We will not be using data source as a criteria for exclusion.

*While I am not aware of any RCT that has used GBTM in its modeling for adherence to date, I would not have excluded them outright. The authors should at a minimum provide how many studies were excluded because they were RCTs.*

Response

Thank you for this suggestion and upon reflection we agree. However, it is important to note that the study abstract would need to state that some form of longitudinal (GBTM, latent class, patterns) analysis on the basis of adherence behaviour was performed in order to be included. As such we have amended the inclusion criteria as follows: ‘Randomised controlled trials will be included if it specified in the study abstract that longitudinal analysis of adherence was performed as part of the study.’

*Similarly, it would also be useful to classify whether GBTM has exclusively been used as the way of classifying adherence as an outcome, or whether it has been applied (and how it has been applied) as an exposure or covariate.*

Response:

It must be stated in the abstract that GBTM (or some variant) has been used to model medication adherence, otherwise it would not be possible to complete the review efficiently. To ensure this is communicated clearly, we have amended the inclusion criteria to the following: ‘Furthermore, publications will have to include in their abstract…’

We agree regarding the use of GBTM and have amended the ‘charting the data’ section to include the following: ‘. Next, information on the study design………., whether adherence was modelled as an exposure, covariate or outcome,’

# Peer review: Reviewer response for version 1

**DOI:** 10.21956/hrbopenres.14152.r27426

**Published:** 2020-05-29

**Authors:** Hannah Durand

**Affiliations:** 1 School of Psychology, National University of Ireland, Galway, Ireland

This article outlines the protocol for a scoping review of group-based trajectory modelling (GBTM) in medication adherence research. This review will make an important contribution to the literature given the vital shift in focus towards longitudinal adherence research that better accounts for variance in patient adherence behaviour over time and disease trajectory. I look forward to seeing the results of this work.

 The authors present a clear rationale for the review. Given the rapid increase in the use of GBTM in this area, this review is timely and well-justified. The protocol is well-written, clear, and detailed. The authors clearly outline the study objectives, specifically to describe the nature, number, scope, and methodology of published research articles using GBTM to measure medication adherence and to identify future research needs.

 The authors propose a scoping review methodology given the broadness of the research question/aims and the considerable heterogeneity of medication adherence research with regard to measurement of adherence behaviour. This measurement heterogeneity is a vitally important consideration in any synthesis of adherence research. This approach to synthesising this particular body of evidence is appropriate. The authors utilise best-practice guidelines for the conduct and reporting of scoping reviews (e.g., Joanna Briggs Institute, 2019; Tricco *et al*., 2018).

 The authors provide sufficient detail on the inclusion and exclusion criteria; the search strategy including databases to be screened, the search string to be used, and details of citation search; the screening procedure; data charting; and on collating, summarising and reporting of results to allow replication.

**Additional minor comment:**

 In line 1 of the abstract, abbreviation GBTM should appear after the word ‘modelling’.

Is the study design appropriate for the research question?

Yes

Is the rationale for, and objectives of, the study clearly described?

Yes

Are sufficient details of the methods provided to allow replication by others?

Yes

Are the datasets clearly presented in a useable and accessible format?

Not applicable

Reviewer Expertise:

Medication adherence; quantitative methods; evidence synthesis.

I confirm that I have read this submission and believe that I have an appropriate level of expertise to confirm that it is of an acceptable scientific standard.

# Response

**Published:** 2020-07-25

**Authors:** Caroline Walsh

**Affiliations:** Royal College of Surgeons, Ireland

We would like to thank the reviewer for reading the manuscript and providing critical analysis. We have updated the manuscript to incorporate the abbreviation suggestion.
